# A realist evaluation of a collaborative model to support research co-production in long-term care settings in England: the ExCHANGE protocol

**DOI:** 10.1186/s40900-021-00257-2

**Published:** 2021-03-20

**Authors:** K. Wilkinson, J. Day, J. Thompson-Coon, V. Goodwin, K. Liabo, G. Coxon, G. Cox, C. Marriott, I. A. Lang

**Affiliations:** 1 University of Exeter, College of Medicine and Health, St Luke’s Campus, Heavitree Road, Exeter, EX1 2LU UK; 2 Classic Care Homes (Devon) Ltd, Pottles Court, Days Pottles Lane, Exminster, Devon, EX6 8RL UK; 3 Southern Healthcare (Wessex) Ltd, Sefton Hall, 11 Plantation Terrace, Dawlish, Devon, EX7 9DS UK; 4 The Peninsula Public Engagement Group (PenPEG) member, University of Exeter, College of Medicine and Health, St Luke’s Campus, Heavitree Road, Exeter, EX1 2LU UK

**Keywords:** Knowledge brokering, Knowledge mobilisation, Implementation, Collaboration, Care homes, Evidence-informed practice, Realist evaluation, Wellbeing

## Abstract

**Background:**

Collaborative working between academic institutions and those who provide health and social care has been identified as integral in order to produce acceptable, relevant, and timely research, and for outputs to be useful and practical to implement. The ExCHANGE Collaboration aims to bring together researchers and people working, living in and visiting care homes to build capacity, share and mobilise knowledge, and identify key areas for future research. This paper describes an embedded, formative, realist and theory-driven evaluation which aims to gather information about how successful the ExCHANGE Collaboration is perceived to be in achieving its aims. An existing realist programme theory from the literature – Closer Collaboration – will be supplemented by two substantive theories: Co-production and Knowledge Brokering. This will result in an initial programme theory which will be tested by this formative evaluation to refine understanding of how the ExCHANGE Collaboration works.

**Methods:**

The evaluation will employ mixed qualitative methods, including: analysis of documents such as feedback forms, Knowledge Broker journal/diary, event attendance records, risk and issues logs and other relevant paperwork gathered as part of project delivery; observations of events/activities; and interviews with care home providers and staff, care home residents, residents’ family members, and researchers who are involved in the project (both project design/delivery, and also attendance or involvement in project activities/events). Framework Analysis will be used to interpret the data collected; analysis will be strategic, by focusing on particular key areas of importance in the developing theory of how the ExCHANGE Collaboration might achieve change.

**Results:**

The results of this study are expected to be published in 2022.

**Discussion:**

This evaluation will investigate how successful the ExCHANGE Collaboration is perceived to be in achieving its aims, in what way, in which contexts, and how this may differ for those involved. It will do this by testing an initial programme theory about how the collaboration works, for whom, under which circumstances, and in what way. Findings will be shared through written publication, an end of project learning event for those involved/interested in the project, and a lay summary to be made publically available.

## Lay summary

This paper describes the planned evaluation of a project currently underway, which aims to support researchers and care homes to work together to identify and carry out research projects. The overarching aim of the collaborative project is to improve care home practices and ultimately residents’ quality of life. By working together, the team will identify best practices for care homes that are more relevant, timely and useful, making it easy to apply in care practice.

The evaluation was designed, and is being delivered by researchers, care home providers and members of the public. It aims to support researchers to learn more about how best to work with care homes, and to provide opportunities for those who manage, work in, live in and visit care homes to learn about how they can use research to improve practice.

The evaluation will regularly assess how successful the project is in achieving its aims in order to learn and adapt as the project progresses. It will also seek to identify whether the activities/events delivered as part of the project are more/less useful for different care homes or individuals, and why. Interviews, observations of project activities and events, and analysis of project documents will form the basis of the evaluation.

The findings of the evaluation will provide useful information about how researchers and care homes can best work together. These findings will be shared through written publications, a learning event and a lay summary.

## Background

Collaborative working between academic institutions and the organisations and individuals who provide health and social care has been highlighted by many as integral to the production of research that is acceptable, relevant, timely, and useful. Co-produced research (that produced in partnership with other professionals/members of the public) has received increasing attention from funders and researchers in health and social care over the past few years [27]; and understanding of how this approach facilitates research use is still developing [12, 14, 28]. Related methods such as participatory action research (involving researchers and participants working together to understand a problematic situation and change it for the better; .e.g. [5, 23]) and community-based participatory research (with the emphasis on researchers joining with the community as full and equal partners in all phases of the research process; e.g. [8, 36]) have been around for some time. These methods involve moving away from hierarchical and instrumental approaches to research and towards more equitable sharing of power, resources, and agenda-setting with an emphasis on social change. The University of Exeter and Care Homes Knowledge (ExCHANGE) Collaboration similarly recognises the importance of creating partnerships between researchers and the people for whom the research is ultimately meant to be of use (i.e. those working in, living in and visiting care homes). Although their roots lie in progressive participatory movements, the values and approaches involved are also reflected in the idea of citizen science [10, 13] and have increasingly been applied in health and social care settings [11, 17, 34].

In health services research another rationale for research co-production is based on the recognition that standard linear approaches (which imply a straight-line from invention and evaluation through to implementation and practical application) are limited in what they can achieve. This recognition has led to the development and funding of approaches that rely more heavily on the involvement and engagement of either patients, family carers and members of the public (patient and public involvement and engagement, PPIE) or of “knowledge users” more broadly. An emphasis on “knowledge users”, which may or may not include patients and the public, is more characteristic of the integrated knowledge translation (IKT) approach developed in Canada [7]. The former approach has grown rapidly in the UK, where state funders such as the National Institute for Health Research [18] and charities such as Alzheimer’s Society [1] have, for several years, required that explicit PPIE be part of all funding bids. Some critics of this approach have contended that a generic PPIE approach ignores issues of power and equality by placing patient and public participants in a subordinate position [15] and moreover ignores the more radical possibilities represented by survivor- and service-user-led research [26]. This is something we hope to address through our approach, detailed below, which brings together multiple groups to decide details of research process and identify the topics in need of research.

Ours is a model in which the interests of all four groups involved - funders, professional researchers, “patients and public”, and people who work in or manage care homes - come together with a shared agenda and a commitment to doing research together. The Care Collaboration Grant programme (2019) [2] was launched by Alzheimer’s Society and The Dunhill Medical Trust. This programme funded three projects to pilot and evaluate creative approaches to collaborating with people working in, living in and visiting care homes. The intention of the projects was to subsequently establish new ways of working collaboratively in the future. The funded projects all relate to research and practice in long-term residential and nursing care (“care homes”) in England, an area in which research and practice gaps have been identified [3, 22, 24] and in which past research has sometimes felt, to those working in the area, to have been done in a top-down way by external “experts” who lack understanding of the realities of living and working in a care-home setting. The ExCHANGE Collaboration is one of these funded projects.

### The ExCHANGE collaboration

The ExCHANGE Collaboration extends ad hoc working that has been taking place for several years between organisations in the south-west of England committed to improving the care and happiness of care home residents. These organisations include: The University of Exeter; The National Institute for Health Research Collaboration for Applied Research Collaboration for the South-West Peninsula (PenARC); The Devon Care Home Collaborative (a group of over 50 independent care providers committed to improving the lives of those living in their care through a programme of continual review and improvement); The South West Academic Health Science Network (SW AHSN). The objective of the ExCHANGE Collaboration is to to build on these existing relationships and develop and test a creative model of engaging care home staff and managers and other stakeholders in research. Importantly, the project does not centre on a single provider but on many small local providers already connected and committed to improvement.

While some of the drivers for involvement in the ExCHANGE Collaboration may differ between academics and care home providers and staff, an agreement on the rationale for the work and an understanding of the contexts within which each organisation functions are essential. Considering this, the ExCHANGE Collaboration activities target learning for both academics and practice. For health and social care staff, activities will aim to develop and sustain capacity for care home managers, staff, residents and their family members to engage with research, understand its meaning and its importance, and be able to apply research evidence to their own practice settings. This understanding of research and its relevance to practice will lead to an increased confidence in applying research in practice; indeed, previous activities delivered by members of this research team to a diverse range of groups including local authorities, patients, members of the public, and police officers and staff have successfully achieved this [35]. For academics, activities will aim to increase their familiarity with practical, political, ethical and emotional factors that underpin the functioning of care homes and engagement with care homes. Both types of activity are key for the ExCHANGE Collaboration’s crucial multi-directional flow of knowledge. The ExCHANGE Collaboration will work to the principle of valuing different kinds of knowledge within a partnership approach.

For the ExCHANGE Collaboration to have lasting impact on improving the lives of care home residents, it needs to be sustainable. In order to do this, and for knowledge to be mobilised, a significant amount of time will be spent building and strengthening relationships with those involved in the ExCHANGE Collaboration and beyond. ‘Knowledge Brokers’ [32] span or bridge the gap between research and practice communities by supporting the exchange of knowledge and values. The idea is that simply by spending time in a setting and “hanging out” there, a Knowledge Broker can engage with and begin to learn what matters to care home providers, staff and residents. The use of a Knowledge Broker working with care homes is novel and within this collaboration is proposed to: (a) ensure that research plans are tailored to focus on improving care-home practice and quality of life experience, and that the latter also drives the former; (b) identify ways of embedding evidence in practice and scaling-up evidence-based improvement; and, (c) engage and involve residents and their relatives who may be unable or unwilling to attend meetings. The ExCHANGE Collaboration will also provide the opportunity for short, goal-oriented placements for interested care-home staff within the University (and vice versa) to facilitate two-way knowledge sharing.

The identification of clear, answerable, feasible, acceptable, and potentially scalable research ideas and implementation problems for which further funding can be obtained is also important for the continuation of the ExCHANGE Collaboration beyond the current funding. Project generation meetings will establish local priorities for a research agenda based on the experience of running similar processes in PenARC (e.g. [33]). These meetings will be used to identify specific issues of concern for those working and living in care homes and other stakeholders (such as primary care practitioners and local authority staff) in the South West and translate these into clear, answerable, and fundable research questions or implementation projects. They will enable the co-design of research projects involving care-home staff and residents, community stakeholders (including family members of residents), and researchers that are flexible and responsive to the needs and expertise of the groups involved.

The ExCHANGE Collaboration therefore has three core aims:

1. To develop and sustain capacity amongst care home managers, staff, residents and family members to engage with, understand, and use research.
2. To mobilise knowledge: that is, to open channels of multi-directional knowledge flow between the partners in the ExCHANGE Collaboration and other relevant stakeholders.
3. To identify feasible, acceptable, and scalable research and implementation problems that are relevant to the needs of people living and working in or visiting care homes, and for which further funding can be obtained.

The ExCHANGE Collaboration model is based on that employed by PenARC, a programme of health and care research intended to promote the translation of knowledge into routine practice underpinned by the theory of Closer Collaboration [12]. Closer Collaboration theory states that where research and implementation projects are co-produced by researchers and end users, they are more likely to generate new evidence or to adapt existing evidence in a way that meets the needs of the end users. As such, the ExCHANGE model of collaboration also draws on Co-production theory [20] to explain how knowledge translation is achieved. Co-production refers to an approach whereby people providing and people receiving services share the power and responsibility, and work together from the start to the end of any project that affects them. To reflect this, the ExCHANGE Collaboration was co-designed with local care home providers from inception. Further, representatives from the owner, staff and resident and family member groups will be present on the Delivery and Management groups in addition to academic researchers and care home provider partners. This means that stakeholders’ input is integral to the decision-making informing the project’s delivery and reporting, all the way through the project lifetime. Individuals from these groups will also be invited at other points in the project to provide input into the development of research materials including data collection tools, and in interpreting and presenting research findings.

The ExCHANGE Collaboration will take into account theories of Knowledge Brokering [32] in achieving successful collaborations. Embedded research models, which describe embedded researchers of some type or another (researchers-in-residence, knowledge brokers), have successfully been used in other contexts (e.g. [29, 37]), and we intend to apply this model in care home settings. These models identify the value of the approach to: (a) ensure that research plans and evidence address and inform care-home practice and working lives and quality of lived experience; (b) identify ways of embedding evidence in practice; and, (c) engage and involve people who live or work in or visit care homes who may be unwilling or unable to attend outside meetings. Embedded research models have proven successful in various ways but previously identified challenges fall into three groups: (1) difficulties related to building relationships, which can be time- and energy-consuming; (2) being able to define and adapt the scope of projects and the need to be flexible while managing competing sets of expectations; and (3) maintaining academic professional identity and attending to the risk of becoming too integrated in the given setting (‘going native’) while having a dual affiliation [9, 30, 31], each element of which can make it harder to avoid becoming biased and losing independence [9, 16].

### ExCHANGE evaluation

Our evaluation aims to understand if and how the ExCHANGE Collaboration achieves the overarching project aims detailed above. The study described in this protocol is a small-scale, embedded evaluation. This will be theory-driven, and will take a formative, realist approach [4, 21] in order to enable real-time feedback to support ongoing amendments to the model. Realist evaluation refers to an approach whereby we will attempt to answer questions such as what works, for whom, in which circumstances, and why? The evaluation will gather information on how successful the ExCHANGE Collaboration is perceived to be in achieving its aims, in what way, in which contexts, and how this may differ for those involved. As mentioned above, the ExCHANGE Collaboration will employ the mechanisms described in the Closer Collaboration model to achieve its overall aims and objectives and our evaluation will explore how they worked in this model. Our evaluation will also explore what helped and hindered the Collaboration and the implications for refining Co-production theory.

Further evaluation of how researchers-in-residence or knowledge brokers can be used effectively in a range of settings is needed and this study may afford opportunities to provide this. Our evaluation will therefore capture the activities of the Knowledge Broker, how relationships are developed, the consequences of these interactions, and the experiences of the Researcher themselves in undertaking this role and the implications for theories of Knowledge Brokering.

## Methods and design

### Objectives

Our evaluation aims to understand the success of the ExCHANGE Collaboration in achieving the overarching project aims detailed above. We will adopt a theory-driven realist approach using qualitative methods [21] to examine in what circumstances and how the model of collaboration works, and for whom. The realist approach enables ongoing adjusting of the underlying theories and their associated mechanisms.

The evaluation will aim to address the following research questions:

- How does the ExCHANGE Collaboration develop capacity, under which circumstances, and for which care home and research staff?
- How does the ExCHANGE Collaboration mobilise knowledge, under which circumstances, and for which care home providers?
- How does the ExCHANGE Collaboration identify research ideas and implementation problems for future investigation, under which circumstances, and for which care home providers?
- What are the implications for theorising how academic-practice collaborations work, under which circumstances, and for whom?

Our evaluation of the study does not seek to be summative and so will not focus on assessing if it ‘works’ as a collaboration programme; rather we seek to generate insights and learning that can be used locally and more widely to others attempting similar collaborative research initiatives.

### Theoretical framework

A realist evaluation approach assumes that programs are “theories incarnate”: that is, whenever a program is implemented, it is testing a theory about what ‘might cause change’ [21]. This evaluation will test and refine an initial programme theory of how the ExCHANGE Collaboration ‘might cause change’. The research team will bring existing theories from the literature (Closer Collaboration, Co-production, Knowledge Brokering) to provide an initial idea (programme theory) about how the collaboration may achieve its aims. This will then be shared and discussed in a team meeting (which will include all key stakeholders) to capitalise on researcher, residents’ family members, and care home staff knowledge and expertise to adapt these theories to this specific ExCHANGE Collaboration. The resulting ‘initial programme theory’ will guide data collection and analysis to understand how contexts may influence which mechanisms work, or not, to produce outcomes.

### Study setting

The evaluation will be carried out over the course of the 24-month project by the research team delivering the wider ExCHANGE Collaboration project. The project will be co-ordinated within the University of Exeter College of Medicine and Health, which supports applied health research and implementation projects. Data collection will take place remotely, via phone or video call, or in person at the University of Exeter or one of the participating care homes in Devon.

### Patient & Public Involvement

Care home residents’ family members and residents themselves (where appropriate) will be supported to engage and contribute to the wider ExCHANGE Collaboration in addition to the evaluation. Two representatives will be invited to join the ExCHANGE Collaboration Management and Delivery Groups and be involved in the decisions that affect the design and delivery of the collaborative model, and the subsequent analysis and dissemination of its findings; through these meetings, the evaluation of the overall project will also be managed. Evaluation tools will be shared with the Delivery Group prior to use and reviewed to ensure they are appropriate for the audience.

The project team will follow the UK National Standards for Public Involvement (NIHR, 2018) [19], including payment and reimbursement of expenses for patient advisors. In line with these, information and support will be provided to the family member(s)/residents(s) to enable their participation, such as holding pre-meetings before Delivery Group meetings to go over the agenda and explain any technical aspects which will aid involvement. Standard two of the document, about ‘working together’, underpins our principle of valuing different kinds of knowledge that people bring to the ExCHANGE Collaboration meetings and workshops. All communication will be in Plain English, and PPI representatives will both be encouraged to contact the researchers directly if anything is unclear, and approached by the project researcher to ensure they are fully able to participate in meetings and workshops. PPI representatives are involved in the governance of the project and its evaluation, by sitting on the project delivery group.

### Data collection

**Fig. 1:**
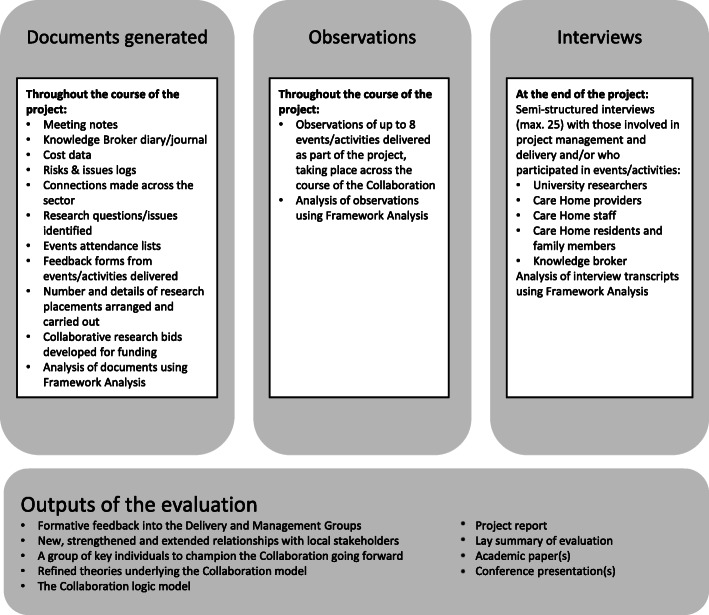
Study flowchart

The evaluation will employ mixed qualitative methods, including observations of events/activities, interviews and analysis of documents such as feedback forms, a Knowledge Broker reflective journal/diary kept by the Researcher (a semi-structured document for Research field notes made following activities, also including reflections on how activities were received etc.), event attendance records, risks and issues logs and other relevant paperwork gathered as part of project delivery. Care home providers, staff, residents, family members and University researchers who are involved in the project will be invited to take part in a short structured interview – this includes those involved in project design and delivery as well as those attending events or taking part in other project activities (see Fig. 1). All data collection tools will be designed by the Knowledge Broker and reviewed by the project Delivery group.

#### Documents generated

A range of documents useful for the evaluation will be gathered and stored electronically (see Fig. 1). Project management documents will be created and updated at regular intervals by the study team. Documents seeking anonymous feedback about how well a project activity/event was received by participants will be developed in consultation with the Delivery Group and handed out after each event. Records of project ideas generated during the project and connections made will also be recorded and regularly updated by the project team. Further, the Knowledge Broker will keep a journal for the course of the project, noting reflections as well as personal experience and learning regarding the role of the knowledge broker and the barriers and facilitators of working in this way.

#### Observations

Observations will be concerned with activities such as learning events and Project Generation Meetings. In addition to researcher observations, project PPI representatives will also be invited to take notes to add to the evaluation data collected and to support the interpretation of events. We have not pre-specified events to be observed because we want to ensure representation of those delivered across the course of the ExCHANGE Collaboration and to allow for the evolving formative model. We will use field notes to record (on paper) the roles of those who were in attendance, the purpose of the activity/event, the focus of discussions, any feedback received verbally or in writing from those involved, and the reflections of the observer on the success of the activity in achieving its goal(s). Prompts focused on refining the programme theory and mechanisms will also be added as the evaluation progresses.

We will seek consent using an opt-in approach whereby attendees will be provided with a consent form at the start outlining what participation involves. For anyone who does not opt in, we will refrain from gathering any information from them or about their participation in the event/activity being observed. Attendees will be able to approach the researcher at any time up to the end of the activity to opt in or out. The researcher will wear a badge so that they can be identified.

#### Interviews

Individual, semi-structured telephone interviews (or face-to face where possible and ethical) lasting up to one hour will be conducted by the project Researcher, with:

- Between 5 and 10 care home staff who have been involved in one or more activities/events held as part of the ExCHANGE Collaboration.
- Between 2 and 5 care home residents or family members who have been involved in one or more activities/events held as part of the ExCHANGE Collaboration or who have been involved in the design and/or delivery of the project (e.g. through involvement with the Delivery Group).
- Between 3 and 5 staff involved in the design and delivery of the project.
- Between 2 and 5 research staff who have been involved in one or more of the project’s activities/events as recipients.

Participants will be identified following their involvement in project activities. Individuals will be invited to take part directly by the Researcher via their preferred communication method (as outlined in their registration for the event/activity they took part in). Where individuals are unwilling or unable to take part, alternatives will be approached until the target number has been achieved. Residents and family members will be recruited independently. We will ensure that the study materials and the interview questions are accessible as recommended by the Enabling Research in Care Homes (ENRICH) network [6]. Where it is not clear that the resident would be able to provide consent, they will be excluded from the research.

Potential participants will be given an information sheet about the evaluation and what the interview will involve, and will be given at least 24 h to decide if they would like to take part. If they agree, informed consent will be obtained. This will make clear that taking part is voluntary and they can choose not to take part without any disadvantage or giving a reason. Where residents or other individuals may require assistance to participate, this will be managed on a case-by-case basis, but participation will be supported wherever possible. If individuals appear distressed during the interview, they will be asked if they wish to stop or take a break. The researcher will let the relevant care home staff know if a resident was tired or distressed.

Interview schedules will be informed by the developing programme theory to learn more about mechanisms, contexts and outcomes of particular interest, and to explore gaps in the emerging theory where more information is needed. This will include: individuals’ experience of the project activities; views on the value of the activity, how well it achieves its aims, how well it was organised and delivered, and any improvements that can be made; views about the ExCHANGE Collaboration model itself – how well it was designed, managed, adapted and delivered over the course of the project – as well as any learning identified, including reflections on the role of the Knowledge Broker and applicability of other programme theories; and the overall barriers and facilitators noted to deliver the model. Prompts or questions focused on refining the programme theory and mechanisms will be added as the evaluation progresses.

For the interviews, each participant will be assigned a participant identification number for all files and transcripts. This number will be recorded on the consent form which will be the only point that links to the participant’s personal details. The interviews will be digitally recorded using an encrypted recording device or by handwritten notes and as soon as possible after uploaded or scanned, following which the recording/notes will be destroyed. Transcripts will be done by a professional transcription service for which a Non-disclosure Confidentiality Agreement and formal contract will be drawn up by the University of Exeter Legal Services Department to ensure that appropriate standards for data handling and transfer are met. Transcripts will be reviewed against the recording for accuracy.

### Data anonymization, storage and analysis

Data will be gathered, recorded, coded and analysed by the study researcher (KW), with support from the project manager (JD) and other members of the Delivery Group, where necessary. All notes and collected data will be pseudonymised and later anonymised, and stored electronically on a University computer that is encrypted and password-protected. Paper notes will be destroyed once typed up.

In analysing the data we will use Framework Analysis, a matrix-based method for ordering and synthesising data [25] that facilitates rigorous and transparent data management and analysis. We will develop a thematic coding framework informed by the initial programme theory and use this to classify and organise the data. Our analysis will be strategic and focus on areas that seem important in relation to the developing theory of how the ExCHANGE Collaboration might achieve change. To increase rigour, coding and interpretations will be discussed between the researcher, project manager and chief investigator (IL), as well as key stakeholders involved in the project. Findings will also be sense-checked with the family members who are part of the project Delivery group, and where possible, we will seek feedback from residents via these family members, or via staff working in the care homes owned by our provider partners. The final step in our analysis will be to consider what our findings from this evaluation add to the existing theories of Closer Collaboration, Co-production and Knowledge Brokering.

We will anonymise quotes or data used in dissemination outputs (i.e. reports, journal publications, presentations, oral feedback/presentations and lay summaries) to ensure that participants and participating organisations cannot be identified, and we will ask respondents before using any quotes from them in these outputs.

## Discussion

### Risk management

We will keep a risk log throughout the course of the evaluation, along with an issues log, to ensure timely and effective resolution to any problems that are identified during the course of the study, as well as providing a narrative to support programme development and evaluation. The log will be maintained by the researcher and project manager, and reviewed by the project team at regular meetings. Risks to the completion of the evaluation as planned are likely to include the absence of key staff involved in the delivery of the study, competing priorities for the care homes involved, and public health concerns or restrictions impacting on the delivery of the ExCHANGE Collaboration model and associated evaluation, particularly in light of the COVID-19 global pandemic. Further risks related to the conduct of the evaluation are outlined below, along with our planned activities to mitigate these risks.

#### Safeguarding

Prior to the start of the evaluation, we will identify any potential safeguarding risks of which members of the research team or care home managers are aware. We will agree protocols and ground rules to follow if problems or safeguarding issues arise (e.g. if poor practice is observed, study findings highlight problems, or if staff or residents identify areas of concern). We will follow the recommendations of the Enabling Research in Care Homes (ENRICH) [6] for concerns about care. If bad practice or something unsafe is observed then the local policy and procedures on adult safeguarding will be followed. Serious concerns about safeguarding will be raised with the local safeguarding service or with CQC.

#### Lone working

This study may involve the researcher working on sites away from the University. We will complete a lone-working risk assessment and any hazards or risks will be identified and managed as per University protocols. We will follow the University of Exeter’s lone-working standard and put appropriate procedures in place to monitor the researcher’s wellbeing. Regular contact at pre-agreed intervals (e.g., when arriving at the site, every hour, and leaving the site) between the researcher and a nominated individual will be made using phones. The nominated person will have copies of the details for all the sites (e.g. phone number and address). We will liaise with care home managers prior to site visits to identify any potential risks for the researcher or anyone else.

#### Conflict of interest

There is overlap between the researchers involved in the delivery of the ExCHANGE Collaboration project and those undertaking this evaluation. To mitigate any bias that might arise we will involve project researchers and management, as well as individuals who have been involved in the project in some way including care home staff, residents and family members, in the overall delivery and monitoring of the evaluation. The evaluation documents as well as the interview questions have been reviewed by members of the Peninsula Patient and Public Engagement Group who have no connection to the ExCHANGE project. Independent researchers at the University have also reviewed the documentation. We will store and analyse information gathered as part of the evaluation separately from any other data relating to the project. We will make clear to individuals resident in care homes owned by members of the ExCHANGE project team that participation in the research is voluntary and no decision they take in relation to this will affect the care they receive or their legal rights in any way.

### Contribution of the evaluation

The findings of our evaluation will provide insight into how researchers and those working, living in, and visiting care homes can best work together and will outline implications for academic-practice collaborations. This will include adaptations to the initial programme theory, which drew upon Closer Collaboration theory, in addition to Co-production and Knowledge Brokering theories of collaborative working, including information about how it works, for whom, under which circumstances, and in what way. Gathering information via different methods, and including researcher reflection (knowledge brokering) in addition to participant feedback (e.g. event feedback forms and interviews) and researcher and family member/resident reflections on the design and running of the ExCHANGE Collaboration will enable a full and comprehensive evaluation of the model. The evaluation will support those attempting to form similar collaborations with care homes in the future.

Findings from the evaluation will be shared with the ExCHANGE Collaboration overall project findings in a number of ways, which will be discussed within the project group, and are likely to include:

- Through written publications (a final report and one or more published papers in peer-reviewed journals).
- An end-of-project learning event for anyone involved or interested in the study.
- A lay summary that will be made available to all participants as well as being made freely available on the University of Exeter College of Medicine and Health website.

### Project timetable

 Ethical approval for the study was received on 31/07/2020 from the Health Research Authority Social Care Research Ethics Committee (Reference: 20/IEC08/0021). Data collection is ongoing. The expected date of completion is July 2022.

## Data Availability

Not applicable.

## Abbreviations

ExCHANGE: The University of Exeter and Care Homes Knowledge Collaboration
PenARC: Peninsula Applied Research Collaboration
NIHR: National Institute for Health Research
PPIE: Patient and Public Involvement and Engagement
ENRICH: Enabling Research in Care Homes
CQC: Care Quality Commission
